# Financial determinants of effective hypertension and diabetes care in rural primary health facilities in Kisumu, Kenya: a mixed-methods study

**DOI:** 10.1186/s12889-026-26963-8

**Published:** 2026-03-11

**Authors:** Nichodemus Werre Amollo, Japheth Ogol, Elijah Museve, Jane Adhiambo Owenga, Dickens Omondi Aduda, Daniel Onguru

**Affiliations:** 1 School of Health Sciences, Jaramogi Oginga Odinga University of Science and Technology, Bondo, Kenya; 2 Department of Family Medicine & Population Health (FAMPOP), University of Antwerp, Wilrijk, Belgium; 3 School of Business and Economics, Jaramogi Oginga Odinga University of Science and Technology, Bondo, Kenya

**Keywords:** Health financing, Primary health care, Noncommunicable diseases, Hypertension, Diabetes, Devolution, Kenya, Financial autonomy, Medication stockouts

## Abstract

**Background:**

Noncommunicable diseases (NCDs), including hypertension and diabetes, account for approximately 27% of all deaths in Kenya, with 26% of adults having elevated blood pressure. Despite devolution of health services to county governments in 2013, financing for NCD management at the primary health care (PHC) level remains weak. This study examines financial determinants shaping hypertension and diabetes care in PHC facilities within a devolved county health system in rural Kisumu County, Kenya.

**Methods:**

We conducted a convergent parallel mixed-methods cross-sectional study in seven public PHC facilities in Seme Sub-County, providing new facility-level evidence on how the interaction between devolution’s financing architecture, facility-level financial autonomy constraints, and resource allocation mechanisms shapes chronic disease care effectiveness in rural Kenya. Quantitative data were collected via structured questionnaires and retrospective document review of financial records (January–August 2024). Qualitative data were gathered through key informant interviews (*n* = 7) with facility in-charges exploring planning, budgeting, and resource allocation. Descriptive statistics were produced in STATA v16; qualitative data were analyzed thematically in R.

**Results:**

All seven facilities prepared annual workplans and budgets, but none achieved comprehensive NCD-specific planning (workplan + budget + dedicated NCD budget line). Funding sources were narrow: 71.4% (*n* = 5) of the facilities depended on NHIF reimbursements and donor support, while only 28.6% (*n* = 2) received direct county funding; 57.1% (*n* = 4) of the facilities relied on only two funding streams. Although all facilities held bank accounts, none had formal financial autonomy and expenditures required county-level approval, typically taking 3–4 weeks (57.1%, *n* = 4) to over two months (28.6%, *n* = 2). Combined with unreliable central supplies, this lack of autonomy meant facilities could not procure locally when stockouts occurred; consequently 85.7% (*n* = 6) of the facilities reported frequent medication stockouts. Facility in-charges attributed these failures to inadequate, unpredictable funding and centralized approval processes that prevented timely local procurement.

**Conclusions:**

Rural PHC facilities operate under structural governance failures in Kenya’s devolved health financing system that systematically undermine effective NCD care. The centralization of financial authority at county level, absence of ring-fenced NCD budgets, and misalignment between planning processes and resource allocation represent system-level policy contradictions rather than facility-level operational deficiencies. Addressing these governance failures requires not only increased funding but constitutional fiscal decision-space for facilities, mandatory NCD budget protection, and reformed disbursement mechanisms essential for equitable chronic care under Kenya’s UHC agenda. The sustainability of chronic care depends fundamentally on facility decision space, not only on funding volume. These findings are transferable to other Kenyan counties under the same devolved framework and to decentralized health systems in sub-Saharan Africa facing similar tensions between fiscal accountability and operational autonomy for chronic disease management.

**Supplementary Information:**

The online version contains supplementary material available at 10.1186/s12889-026-26963-8.

## Background

Noncommunicable diseases (NCDs) constitute a rapidly escalating public health crisis in sub-Saharan Africa, with hypertension and diabetes representing particularly urgent challenges. Globally, NCDs account for over 70% of all deaths, disproportionately affecting low- and middle-income countries (LMICs) [1, 2]. Kenya is experiencing a growing NCD burden; while NCDs collectively account for 27% of all deaths in the country, cardiovascular diseases are a major contributor, responsible for roughly 13% of all deaths according to recent estimates [3]. National surveys indicate that approximately 26% of adults have elevated blood pressure and 5.6% have raised blood glucose levels [4, 5], creating a substantial and growing demand for chronic disease management services.

Effective management of hypertension and diabetes requires sustained access to screening, diagnosis, treatment, and long-term monitoring with affordable medicines [6, 7]. Delivering these services depends critically on adequate and predictable health financing [8, 9]. In this study, effectiveness is operationalized in line with the Kenya Health Financing Strategy (2020–2030), which identifies effectiveness and efficiency as core strategic principles guiding health-financing reforms [10, 11]. Effectiveness reflects the extent to which financing mechanisms enable facilities to achieve desired health outcomes through: (1) continuous availability of essential medicines and diagnostics; (2) continuity of patient care and follow-up; (3) prevention of severe complications requiring hospitalization; and (4) responsiveness to patient needs. Other guiding principles such as appropriateness, transparency, social solidarity, and equity are evaluated in this study within the context of NCD financing at the primary health-care level.

According to the World Health Organization’s health-financing framework, the capacity of a health system to deliver quality, accessible services without financial hardship depends on how resources are raised/collected, pooled, and used to purchase services [10, 11]. In Kenya, health financing functions through a mix of mechanisms aimed at achieving Universal Health Coverage (UHC). Sources include government tax-based allocations (national and county), donor contributions, out-of-pocket payments, and insurance schemes [10]. However, the flow and management of these resources at the facility level largely determine service delivery capacity, particularly for chronic conditions that require sustained care [9, 12].

Primary health care (PHC) facilities form the foundation of Kenya’s health system under the Kenya Essential Package for Health (KEPH) framework. The framework defines a tiered system of increasing service complexity: Level 1 (community health units) provide health promotion and referral linkages; Level 2 (dispensaries) offer basic outpatient care and NCD screening; Level 3 (health centres) provide expanded outpatient, laboratory, and maternal care; and Level 4 (sub-county hospitals) deliver comprehensive services including inpatient and specialized NCD management. Each successive level requires greater financial and human-resource inputs, with financing expected to align proportionately with function. However, PHC facilities (Levels 2–4) often operate under resource constraints that limit their ability to provide consistent NCD services [13–16].

Kenya’s health sector underwent major transformation with the 2010 Constitution and the 2013 devolution reforms, which transferred governance authority from the central government to create a two-tier system comprising the national government and 47 semi-autonomous county governments [17]. Counties assumed responsibility for delivering services at community and primary levels, while the national government retained policy and regulatory oversight [11]. Devolution was intended to enhance responsiveness, accountability, and efficiency by bringing decision making and resources closer to populations [14, 17, 18]. However, in practice, the fiscal and administrative rules that govern public funds have often limited facility level decision space, contributing to fragmented planning, delayed and unpredictable disbursements, and wide variation in county prioritisation of primary care services [16, 18–20]. Although health sector allocations increased from 6% of the national budget in 2015 to 11% in 2023 [18–21], these remain below the 15% Abuja target and are insufficient to meet the growing burden of chronic disease.

A pivotal reform shaping facility financing within devolution was the Public Finance Management Act of 2012 [22]. Prior to this, the Health Sector Services Fund enabled many primary facilities to retain and manage funds locally through facility bank accounts and committees, which improved flexibility to address operational needs and procure essential supplies when central supply was delayed or insufficient [23]. Following implementation of the Public Finance Management Act, most counties required facility revenues and operating funds to flow through county treasuries, with expenditures requiring county level approval. While intended to strengthen accountability, these arrangements often reduced the timeliness and flexibility of spending decisions at facility level, which is particularly consequential for hypertension and diabetes services that depend on continuous medicine availability, functioning diagnostics, and rapid response to stockouts and equipment failure [17, 21, 24].This creates a political institutional gap in Kenya’s devolved health system, where service delivery responsibility is decentralised but practical fiscal decision space for frontline facilities remains constrained.

Financing constraints at the facility level have direct implications for NCD service delivery. Inadequate and unpredictable funding compromises the consistent availability of medicines, diagnostics, and trained personnel, all vital for effective chronic care [16, 22, 25]. Unlike vertical programs for HIV, TB, and malaria that benefit from earmarked funding, NCD programs lack ring-fenced budget al.locations and are integrated into general PHC budgets, leaving them vulnerable to competing priorities. Limited data on NCD burden, cost, and utilization further weaken their case during budgeting cycles [15, 24]. Consequently, NCD services frequently suffer from chronic underfunding and irregular supplies of essential drugs.

Non-state actors contribute variably to NCD financing at the county level. Development partners such as Danida have supported selected counties, while social health insurance reimbursements through NHIF/SHA offer limited financial protection. However, delays in reimbursements and unpredictable payment amounts continue to destabilize facility operations for chronic disease management [13, 23, 26].

These systemic financing gaps have significant implications for Universal Health Coverage (UHC) and health equity. Effective UHC requires that all individuals, regardless of socioeconomic or geographic status, have access to quality health services without financial hardship. Yet, the current financing architecture in Kenya’s devolved system limits equitable access to essential chronic disease services particularly in rural areas where facilities depend on narrow, unpredictable revenue streams and have limited fiscal autonomy.

This study therefore examines how the interaction between financing architecture and constrained facility decision space under devolution shapes the effectiveness of hypertension and diabetes care at primary health care level. Despite multiple financing reforms and the growing burden of NCDs, empirical evidence on how financing mechanisms at the PHC facility level affect hypertension and diabetes service delivery remain limited. Previous studies have addressed health-system financing broadly [14, 21, 24], but have rarely examined facility-level processes or incorporated managerial perspectives. Understanding how revenue sources, budgeting processes, and resource allocation decisions interact to shape service effectiveness is critical for informing equitable health-financing reforms.

This study contributes by providing facility-level evidence on how financing architecture and fiscal decision space interact to shape chronic care continuity in a devolved system. By integrating facility managers’ perspectives with financial data, the study clarifies mechanisms through which governance arrangements affect service delivery effectiveness for hypertension and diabetes at primary health care level.

Kisumu County in western Kenya offers a relevant context for this inquiry because recent research indicates a substantial noncommunicable disease burden and important gaps in primary health care capacity for hypertension and diabetes management [27]. Evidence from care seeking populations in Kisumu similarly shows a high hypertension burden. In a study conducted among adults seeking care at Kisumu County Hospital, the overall prevalence of hypertension was 22% [28]. Against this backdrop, this study investigates how financing arrangements influence effective hypertension and diabetes management in Seme Sub County primary health care facilities. Specifically, the study aims to: (1) identify sources of funding for noncommunicable disease management; (2) examine planning and budgeting processes; (3) assess allocation and utilization of financial resources; and (4) analyse how financing mechanisms influence the effectiveness of noncommunicable disease service delivery. Findings aim to provide actionable evidence to inform Kenya’s ongoing pursuit of universal health coverage and strengthen financial resilience within devolved health systems.

## Methods

### Study design

We conducted a convergent parallel mixed-methods cross-sectional study using a multi-site case study approach [29]. Quantitative and qualitative data were collected concurrently, analyzed separately, and integrated during interpretation to generate complementary insights into facility financing mechanisms. The design was QUAL-dominant at the analytical stage because the small facility sample size (*N* = 7) limited statistical inference, while qualitative data provided essential contextual understanding [30]. Both data types were, however, equally valued during data collection and interpretation.

### Study setting

The study was conducted in Seme Sub County, a predominantly rural sub county of Kisumu County in western Kenya. Kisumu County is one of Kenya’s 47 counties and comprises seven sub counties, with an estimated population of about 1.2 million served by approximately 70 primary health care facilities at levels 2 to 4, including dispensaries, health centres, and sub county hospitals [27]. Kisumu County was purposively selected because recent studies conducted in the county have reported a substantial burden of hypertension in care seeking populations and have documented gaps in primary health care capacity for hypertension and diabetes management [28].

Kisumu provides a relevant implementation context because it has been involved in national and county efforts to strengthen primary health care coordination through Primary Care Networks and digital health initiatives. Primary Care Networks in Kenya are organized using a hub and spoke model in which a level 4 facility functions as a coordination hub that supports surrounding level 2 and level 3 facilities and community health units, with the aim of strengthening continuity of care through structured referral and counter referral across levels of care [31]. Kisumu has also been an early implementation county for the electronic Community Health Information System. eCHIS deployment began with a pilot in Kisumu County in 2021 followed by national rollout, and county planning documents describe digitization efforts aimed at improving information flow and coordination between community and facility platforms [32]. Seme Sub County was purposively selected as a predominantly rural setting and because study implementation was feasible based on engagement with the county and sub county health management teams and the availability of routine facility financial records required for the assessment.

This research was conducted within the context of the Flemish Interuniversity Council-Institutional University Cooperation (VLIR-IUC) and JOOUST programme, a long term collaboration between JOOUST and Flemish universities that aims to strengthen university capacity in research and training across domains including health [33]. Within this programme, Subproject 3 focuses on building capacity for research, management, and control of communicable and noncommunicable diseases, including type 2 diabetes and hypertension, and strengthening local laboratory capacity [33]. For the current study, this programme facilitated engagement with county and sub county stakeholders and supported access to participating facilities, but it did not influence the study objectives, tool development, data collection, analysis, or interpretation. Data were collected between January and August 2024.

### Study population and sampling

The target population comprised public primary health care facilities providing hypertension and diabetes services in Seme Sub County. In the wider Seme sub county setting, primary health care services are delivered through facilities at levels 2 to 4. In consultation with the Seme Sub County Health Management Team, we identified 8 out of 29 PHC facilities providing both hypertension and diabetes services. All eight public facilities are located in rural catchment areas. Seven facilities ultimately participated after one facility declined due to administrative constraints. The participating facilities included two level 4 facilities, three level 3 facilities, and two level 2 facilities, consistent with the Kenya Essential Package for Health (KEPH) classification.

Facilities were purposively selected using the following criteria: the facility is publicly owned and managed under the county health system, provides services for both hypertension and diabetes, and the facility management team agreed to participate. Although the facility sample was small, it captured the major public primary care facilities providing these services in the sub county across levels 2 to 4. The facilities exhibit typical characteristics of rural primary health care facilities in western Kenya: government-owned, county-managed, serving predominantly rural populations, and operating under similar policy and financing frameworks. These characteristics enhance transferability of findings to similar rural contexts in Kenya, though variations in county governance capacity and resource availability must be acknowledged.

Because this study used a multi-site case study approach, each facility constituted an analytic case. The facility sample was intended to achieve coverage across the public primary care levels that provide hypertension and diabetes services in the sub county and to enable cross case comparison of financing arrangements and decision space across levels 2 to 4. In practice, the sample approximated a census of eligible public facilities in Seme that provided both hypertension and diabetes services during the study period. Seven facilities provided sufficient variation for analytic comparison while remaining feasible for detailed review of routine financial records and in depth key informant interviews with facility in charges.

We conducted one key informant interview at each facility, resulting in seven key informant interviews in total. The key informants were purposively selected based on their roles and direct involvement in financial management or NCD service delivery. Primary informants were facility in-charges, who possessed comprehensive knowledge of facility operations, budgeting processes, and service delivery challenges.

Although the study is not designed for statistical generalisation beyond the seven facilities, it supports analytic generalisability by identifying mechanisms through which financing architecture and facility decision space shape continuity of chronic care. These mechanisms, including predictability of funding, approval turnaround times, and the ability to procure during supply gaps, are plausibly applicable to other rural primary health care settings operating under similar devolved public finance rules and purchasing arrangements.

### Conceptual framework

**Fig. 1:**
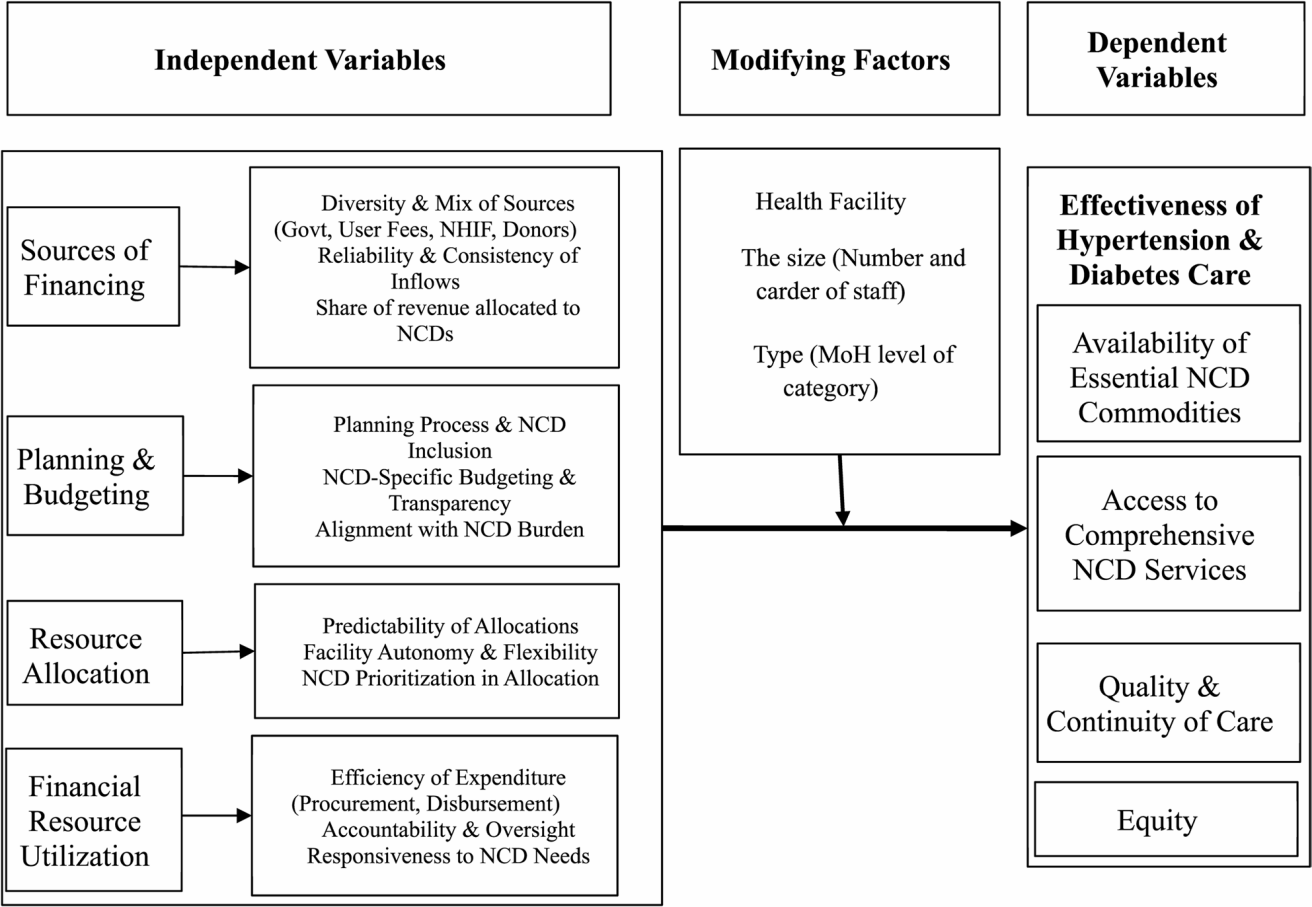
Conceptual framework illustrating the influence of financial mechanisms and contextual factors on hypertension and diabetes care effectiveness in public PHC facilities, seme subcounty

The study was guided by the World Health Organization (WHO) health financing framework, adapted to the facility level. This framework conceptualizes NCD care effectiveness as influenced by four interrelated financial dimensions [23, 34]. Figure 1 illustrates the conceptual framework guiding this study. The framework posits that NCD care effectiveness is influenced by a cascade of financial determinants organized into four interrelated dimensions: (1) sources of financing (diversity and mix of funding sources including government, user fees, NHIF, Facility Improvement Fund (FIF) - a conditional grant designed to support facility-level infrastructure and operational improvements, donors; reliability and consistency of inflows; share of revenue allocated to NCDs); (2) planning and budgeting (planning processes and NCD inclusion; NCD-specific budgeting and transparency; alignment with NCD burden); (3) resource allocation (predictability of allocations; facility autonomy and flexibility; NCD prioritization in allocation); and (4) financial resource utilization (efficiency of expenditure in procurement and disbursement; accountability and oversight; responsiveness to NCD needs). These dimensions are moderated by health facility characteristics (size, staffing, facility type/KEPH level) and collectively determine the dependent variable: effectiveness of hypertension and diabetes care, measured through availability of essential NCD commodities, access to comprehensive NCD services, quality and continuity of care, and equity in service provision.

### Data collection

Data collection occurred between January and August 2024, prior to the full implementation of Kenya’s 2023 Social Health Insurance Act (enacted November 2023) [35]. At the time of data collection, the National Hospital Insurance Fund (NHIF) remained the primary social health insurance mechanism, though the Social Health Authority (SHA) that replaced NHIF had been legally established and was in early implementation phases. The SHA began full operations in October 2024, after our data collection was complete. All data therefore reflect the financing landscape under the NHIF regime and the transition period to SHA.

Interpretation of results should therefore be understood as reflecting facility financing and approval arrangements under the NHIF purchasing environment and county-controlled expenditure authorization practices during the transition period. The findings provide a baseline for assessing whether SHA implementation improves the timeliness of payments, facility liquidity, and the speed of decision making for procurement of essential commodities for hypertension and diabetes care.

### Quantitative data collection

Quantitative data were collected using a structured questionnaire adapted from the WHO Package of Essential Noncommunicable Disease Interventions (PEN) framework [6, 36], and health facility financing assessment tools applied in similar Kenyan contexts [27]. The tool was modified to include additional variables specific to financial autonomy, NCD-specific budgeting practices, and resource allocation mechanisms at the primary health care level, reflecting Kenya’s devolved health financing architecture and the policy context of county-managed health facilities. Modifications included detailed questions on funding source diversity (NHIF, county allocations, Facility Improvement Fund, donor support, user fees), expenditure approval processes and timelines, medication stockout frequency and causes, and perceived effectiveness of different financing mechanisms for NCD care. The final English version of the questionnaire used in this study is provided as a supplementary file (Additional File 1). The tool captured information on facility characteristics, funding sources, planning and budgeting practices, resource allocation, and NCD service availability. Data were collected using KoboToolbox on tablets, enabling real-time validation.

Financial information was extracted through retrospective review of records, including bank statements, budget reports, expenditure ledgers, and procurement receipts, from January to August 2024. Structured interviews with facility in-charges were conducted to clarify incomplete records and validate financial data, especially regarding funding sources and fund-approval timeframes.

### Qualitative data collection

Qualitative data were collected through face-to-face key informant interviews with facility in charges using a semi structured interview guide developed for this study (Additional File 1). The guide was informed by the WHO health financing framework [23, 34] and health facility financing literature in devolved contexts [15, 28, 29], with questions tailored to explore how financing mechanisms specifically affect NCD service delivery in rural primary health care settings. The interview guide explored: (1) sources and adequacy of funding for NCD services; (2) planning and budgeting processes and the extent to which NCDs are prioritized; (3) challenges in fund access, allocation, and utilization; and (4) the perceived impact of financing mechanisms on NCD service delivery quality and patient outcomes. Interviews were conducted in English (the language of professional health care communication in Kenya), lasted approximately 45–60 min, and were audio-recorded with informed consent. The interview guide used open-ended questions such as “Can you describe how your facility plans and budgets for NCD services?” followed by probing questions to explore mechanisms and consequences (e.g., “How does this affect your ability to manage patients with diabetes?“). An observation checklist was also used to systematically document the availability and functional status of essential NCD resources, including medications (specifically checking for common antihypertensives and antidiabetics listed in WHO PEN), blood pressure apparatus, glucometers, test strips, and patient registers/management tools.

### Data management and analysis

#### Quantitative analysis

Quantitative data were entered and cleaned in Microsoft Excel before being exported to STATA version 16 (StataCorp, College Station, TX, USA) for analysis. Given the small sample size (*N* = 7 facilities), analysis focused on descriptive statistics. Frequencies, percentages, means, and ranges were calculated to summarize facility characteristics, funding sources, planning and budgeting practices, financial autonomy indicators, and NCD service readiness measures. Results were tabulated to facilitate pattern identification and comparison.

#### Qualitative analysis

Audio recordings were transcribed verbatim and transcripts were validated against original recordings for accuracy. The transcripts were imported into the R programming language (version 4.2.2) for data management, coding, and documentation of analytic decisions. Text files were imported and organized using the *tidyverse* suite, including *readr* for import, *dplyr* and *tidyr* for structuring transcript level datasets, and *stringr* for text cleaning and standardisation. A text corpus was created using the *tm* package to support systematic handling of documents and retrieval of coded excerpts [37].

Thematic analysis followed the approach described by Braun and Clarke [38]. Coding used a combined deductive and inductive strategy [30]. Initial codes were developed from the conceptual framework domains and interview guide, then refined iteratively as additional concepts emerged from the data. Coded excerpts were stored as structured tables linked to transcript identifiers, and a codebook was maintained to document code definitions, inclusion criteria, and exemplar quotations. Two researchers independently coded a subset of transcripts to refine the codebook and improve consistency, with discrepancies resolved through discussion.

To support theme refinement and provide an additional audit trail, we generated token based summaries using tidytext and tokenizers [39]. Tokenisation outputs were used to examine recurring terms and co-occurrence patterns related to facility financing processes, including approval timelines, reimbursements, autonomy, and medicines availability. These outputs were used to support interpretation and to check that themes were grounded in the textual data, and they were not used as a substitute for thematic coding.

### Data integration

Qualitative findings were integrated with quantitative results produced in Stata during interpretation using triangulation [40]. Facility identifiers were harmonised across datasets, and qualitative themes were compared against facility level descriptive indicators to explain observed quantitative patterns. Integrated findings were summarised through narrative synthesis supported by cross facility comparison and joint presentation of themes alongside descriptive statistics.

The study also identifies facility level indicators that can be tracked in post SHA evaluations, including reimbursement timeliness, predictability of county disbursements, approval turnaround time for facility expenditures, availability of emergency procurement mechanisms, and frequency and duration of stockouts for essential hypertension and diabetes medicines and diagnostics.

### Validity and reliability

To ensure instrument validity, the questionnaire and interview guide were pre-tested at a health facility outside the study sample. Feedback was used to refine question clarity, structure, and cultural appropriateness. Tools were also reviewed by the research supervisory team, comprising experts in health financing and epidemiology, to ensure content validity. For the qualitative component, reliability was enhanced through consistent use of the interview guide, systematic probing techniques, and audio recording to minimize interviewer bias and data loss.

### Ethical consideration and reflexivity

Ethical approval was obtained from the JOOUST Ethics Review Committee (ERC/5/24–06) and a research licence was obtained from the National Commission for Science, Technology and Innovation (NACOSTI/P/23/25192). Permission was also obtained from the Kisumu County Department of Health and the Seme Sub County Health Management Team. All participants provided written informed consent. Interviews were conducted in private spaces at facilities, audio recorded with permission, and anonymised during transcription. Data were stored on password protected devices accessible only to the research team.

The research team comprised investigators with training in health financing and health systems research and familiarity with the Kenyan primary health care context. We recognised that our professional backgrounds and prior engagement with county and facility stakeholders could influence how questions were asked, how participants responded, and how data were interpreted. To minimise these effects, interviews were conducted using a semi structured guide and neutral probing, with emphasis on allowing participants to describe processes and experiences in their own terms. We held regular debrief meetings during data collection to reflect on interviewer positioning, refine interviewing approaches, and document emerging assumptions. During analysis, we maintained analytic memos to record coding decisions and interpretations, and we used team discussions to challenge and refine themes. A subset of transcripts was independently coded by two researchers and discrepancies were resolved through discussion to improve consistency. These steps supported transparency in interpretation and strengthened the credibility of the qualitative findings.

## Results

### Facility characteristics

Seven primary health care (PHC) facilities participated in the study, all located in rural settings (Table 1). Facilities represented a mix of KEPH service levels: two Level 4 sub-county hospitals (28.6%), three Level 3 health centres (42.9%), and two Level 2 dispensaries (28.6%). No Level 1 community units were included because they do not provide clinical NCD care. Most facilities (71.4%, *n* = 5) had at least one staff member trained in NCD management. Although all possessed basic diagnostic equipment for hypertension and diabetes (blood pressure apparatus and glucometers), fewer than half (42.9%, *n* = 3) considered their equipment adequate for patient demand. Only two facilities (28.6%) reported generating internal revenue through user fees for NCD-related services.

**Table 1:** Characteristics of participating primary health care facilities (*N* = 7)

Characteristic	Number (*n*)	Percentage (%)
Facility Level		
Level 4 (Sub- County Hospital)	2	28.6
Level 3 (Health Centre)	3	42.9
Level 2 (Dispensary)	2	28.6
Location		
Rural	7	100
NCD Service Readiness		
At least one staff member trained in NCDs	5	71.4
Has essential NCD diagnostic equipment	7	100
Reports equipment as adequate for patient load	3	42.9
Revenue Generation		
Generates revenue from user fees	2	28.6

### Sources of facility funding for NCD care

The main funding sources were NHIF reimbursements (71.4%, *n* = 5) and Danida support (71.4%, *n* = 5). Only 28.6% (*n* = 2) of facilities received direct county government funds through the Facility Improvement Fund (FIF). More than half (57.1%, *n* = 4) relied on only two funding sources (Table 2).

**Table 2:** Sources of facility funding for NCD care (*N* = 7)

Indicator	Number (*n*)	Percentage (%)
A. Frequency of Funding Sources Mentioned		
NHIF	5	71.4
Danida	5	71.4
County Government / FIF	2	28.6
User Fees	2	28.6
M-PESA Foundation	1	14.3
Other NGO Support	0	0
B. Diversity of Funding Sources per Facility		
One Source	2	28.6
Two Sources	4	57.1
Three or More Sources	1	14.3

*N* = 7 facilities. Sources are not mutually exclusive

Facility in-charges reported that these funds were neither sufficient nor specifically designated for chronic disease management:

*“We cannot specifically say this money is for NCDs. It’s all pooled for many competing needs.”* (In-charge, Level 3 facility).

In-charges from facilities supported by Danida noted that reliance on donor funding created uncertainty, while those receiving NHIF reimbursements cited payment delays of two to four months, limiting planning capacity:

*“NHIF takes months to reimburse—sometimes three or four months. How do you plan with that?”* (In-charge, Level 3 facility).

County government support was described as minimal or irregular:

*“County support is very limited. We depend mainly on what we generate or on NHIF*,* but even those are unpredictable.”* (In-charge, Level 4).

### Planning and budgeting for NCD services

All seven facilities reported preparing annual workplans and budgets, fulfilling administrative requirements (Table 3). However, none demonstrated comprehensive NCD-specific planning, that is, having a workplan, budget, and dedicated budget line for NCDs. While a majority (71.4%, *n* = 5) reported considering NCDs in their budgets, not a single facility (0%) achieved what we defined as comprehensive NCD planning having a work plan, a budget, *and* specific, dedicated consideration for NCDs within that budget.

**Table 3:** Planning and budgeting practices for NCD services (*N* = 7)

Planning & Budgeting Practice	Number (*n*)	%
Facilities with annual work plans	7	100
Facilities with annual budgets	7	100
Facilities that specifically consider NCDs in the budget	5	71.4
Facilities with comprehensive NCD planning*	**0**	**0**

*Defined as having a work plan, a budget, AND specific consideration for NCDs

In-charges reported that planned NCD activities were often not implemented because final allocations and disbursements differed from facility budgets. Several described limited linkage between budgeting and executed funding, particularly in the absence of a dedicated NCD budget line:

*“We prepare very detailed budgets with input from all departments*,* but at the end of the day*,* you get what is available*,* not what you budgeted for.”* (In-charge, Level 3 facility).

*“The process feels like an exercise in futility sometimes. We spend days preparing budgets that are never followed when the actual allocation comes.”* (In-charge, Level 4 facility).

All in-charges emphasized the impossibility of meaningful NCD budgeting given the absence of dedicated NCD budget lines and overall resource scarcity. One in-charge quantified this inadequacy:

*“It doesn’t [work]*,* because you only budget with the money received from the facility. And the money is usually very inadequate. Sometimes you can even give NCDs 2*,*000 [Kenyan Shillings*,* approximately USD 15] for the whole year.”* (In-charge, Level 4 facility).

Most in-charges (6/7) also described limited facility input into final budget decisions despite the participatory process:

*“We participate in making the budget and presenting our needs*,* but the implementation is done by the county. They don’t follow what we proposed.”* (In-charge, Level 2 facility).

However, two in-charges from Level 4 facilities offered a more nuanced perspective. While acknowledging that NCD budgets were inadequate and often not fully actualized, they emphasized that the budget process did allow them to advocate for resources and occasionally influence allocation decisions:

*“It’s not perfect*,* and we rarely get what we ask for*,* but at least we can present what we need. Sometimes we get a portion of what we requested*,* especially if we justify it well with data.”* (In-charge, Level 4 facility).

### Financial autonomy and expenditure control

While all facilities (100%) operated bank accounts, their control over these funds was minimal (Table 4). All seven facilities (100%) required external approval from county health administration to spend funds from their accounts. Consequently, only two facilities (28.6%) perceived themselves as having any degree of spending autonomy. A majority of facilities (57.1%, *n* = 4) reported that the authorization process typically took 3–4 weeks, while for others delays extended to 2–3 months (28.6%, *n* = 2) or even beyond three months (14.3%, *n* = 1).

**Table 4:** Financial autonomy and expenditure control indicators (*N* = 7)

Indicator	Number (*n*)	%
A. Financial Autonomy Indicators		
Facilities with a bank account	7	100
Requires external approval to spend funds	7	100
Report moderate-high perceived spending autonomy**	2	28.6
Has formal financial autonomy*	0	0.0
B. Average Timeframe for Spending Authorization		0
3–4 Weeks	4	57.1
2–3 Months	2	28.6
More than 3 Months	1	14.3

*Formal financial autonomy = legal authority to authorize expenditures without external approval

**Perceived autonomy = in-charges’ subjective sense of control over resource use decisions

***Based on *n* = 7; all facilities require authorization

Two in-charges reporting moderate perceived autonomy felt they could *request* and sometimes influence specific expenditure decisions through advocacy to county officials, even though final approval rested with the county. This contrasted with other in-charges who felt they had no meaningful input into spending decisions:

*“At least we can request what we need and sometimes they listen*,* especially for urgent things. But it’s their decision in the end.”* (In-charge, Level 4 facility).

All in-charges reported that even funds generated locally were deposited in county-controlled accounts and could not be used without authorization.:

*“The money collected through paybill goes to county and then sent to our account*,* but I can’t use it. I have to be authorized to use it*,* which can take weeks.”* (In-charge, Level 4 facility).

*“Even money we generate ourselves through user fees*,* we can’t touch without approval. It doesn’t make sense—we know what we need here.”* (In-charge, Level 3 facility).

All in-charges emphasized that authorization delays created cascading operational problems:

*“The county sometimes waits for other budgets and this makes them take longer to authorize the money. We don’t have direct withdrawal*,* so we just wait.”* (In-charge, Level 2 facility).

Most in-charges (6/7) specifically linked this lack of financial autonomy to their inability to respond to supply chain failures:

*“When KEMSA doesn’t supply and we need to buy locally*,* we can’t act fast. We have to wait for county approval and by then there’s a stockout.”* (In-charge, Level 2 facility).

*“If we had even small petty cash for emergencies*,* we could buy locally when there are gaps. But we can’t do anything without approval*,* so patients suffer.”* (In-charge, Level 3 facility).

### Effects of financing mechanisms on NCD service delivery

A majority of facilities (85.7%, *n* = 6) experienced frequent or very frequent stockouts of essential hypertension and diabetes medicines, with 42.9% (*n* = 3) reporting stockouts occurring almost always and another 42.9% experiencing stockouts more than four times annually (Table 5). Only one facility (14.3%) reported merely occasional stockouts (3–4 times per year).

**Table 5:** Service readiness and medication availability indicators (*N* = 7)

Indicator	Number (*n*)	Percentage (%)
A. Service Readiness		
Facilities with NCD-trained staff	3	42.9
Facilities with adequate NCD equipment	3	42.9
B. Medication Availability		
Reports frequent or very frequent medication stockouts	**6**	85.7
* Very frequently (almost always)*	*3*	42.9
* Frequently (more than 4 times per year)*	*3*	42.9
* Occasionally (3–4 times per year)*	*1*	14.3

In-charges linked these shortages to unreliable central supplies and inability to procure locally:

*“Inconsistent supplies from both the county warehouses and KEMSA [Kenya Medical Supplies Authority]*,* plus inadequate finance to make local arrangements for the drugs when there are gaps.”* (In-charge, Level 2 facility).

### Clinical outcomes and patient safety

All in-charges reported that medication stockouts directly led to disease progression and preventable complications. They described patients presenting with uncontrolled hypertension or diabetes because they could not access medications:

*“We see patients deteriorating because of stockouts. They come back with strokes*,* kidney problems*,* or diabetic complications that could have been prevented if we had consistent drug supply.”* (In-charge, Level 4 facility).

*“[There is] no effective diagnosis and monitoring when equipment breaks down and also misdiagnosis because equipment are faulty but we can’t get them fixed without approval for funds.”* (In-charge, Level 2 facility).

### Patient trust and continuity of care

All in-charges emphasized that persistent service failures eroded patient confidence in the public health system, leading to treatment abandonment and loss to follow-up:

*“When patients come here repeatedly and we have no medicine*,* they lose faith in us. They stop coming for their appointments*,* which leads to poor adherence and worse outcomes. We see them later with complications.”* (In-charge, Level 4 facility).

*“Stockouts and delay in services because of financing problems—clients run away because of these. They go to private facilities or just stop treatment.”* (In-charge, Level 3 facility).

*“Once you disappoint a patient several times*,* they don’t come back. Then we lose them until they have an emergency. That’s not how chronic disease care should work.”* (In-charge, Level 2 facility).

### Staff morale and operational capacity

Most in-charges (6/7) described how financial constraints placed immense strain on health workers, who possessed clinical skills but lacked the resources to apply them effectively:

*“It is demoralizing. You have the skills and training*,* but you cannot offer the service because there are no medicines or the glucometer has no strips. That’s very frustrating for a professional.”* (In-charge, Level 2 facility).

*“We have staff who are overworked and burnt out*,* and at the same time*,* we cannot even find funds for basic things like batteries for the BP machines or glucometer strips. It becomes a constant struggle that affects morale.”* (In-charge, Level 2 facility).

*“Our nurses and clinical officers feel helpless when they can’t help patients because of system failures beyond our control. Some are considering leaving for facilities where they can actually practice.”* (In-charge, Level 4 facility).

## Discussion

This study contributes to comparative debates on decentralisation and chronic care by demonstrating how facility financing architecture and fiscal decision space interact to shape continuity of hypertension and diabetes services in rural primary health care. In decentralised systems, improvements in responsiveness depend not only on transferring responsibilities to local governments, but also on whether frontline facilities have timely access to funds and practical authority to use them for essential commodities and operations. For chronic conditions that require continuous medicines, diagnostics, and follow up, delays in purchasing and constrained spending authority can translate rapidly into service interruptions [6, 7]. Our findings therefore speak to a broader governance challenge in decentralised health systems, namely how accountability arrangements can unintentionally reduce local responsiveness for services that depend on continuity.

A key finding is that facilities reported preparing annual workplans and budgets, yet these processes were often weakly linked to executed allocations and disbursements. In the absence of dedicated budget lines for noncommunicable disease services, facility plans for hypertension and diabetes were frequently subsumed within general budgets and competed with other priorities. Similar fragmentation between planning and budgeting has been documented in other devolved county contexts in Kenya, where facility and sub county plans do not consistently translate into predictable resource flows [15, 41, 42]. For chronic care, this disconnection matters because it undermines the ability to plan procurement, sustain counselling and follow up activities, and maintain minimum diagnostic and medicine availability required for stable disease control [6, 7, 36].

The findings also indicate that the composition and predictability of facility revenue contributed to fragile service readiness. Most facilities relied on a narrow set of funding sources, particularly insurance reimbursements and external support, with limited direct county funding through routine mechanisms. Participants described delays in reimbursements and uncertainty in external support, which reduced liquidity and constrained routine purchasing decisions. These patterns align with broader evidence that noncommunicable diseases receive comparatively limited external assistance, raising concerns about sustainability where domestic financing and purchasing arrangements remain weak [43]. In this context, strengthening chronic care requires a financing approach that increases predictability and reduces volatility in facility operating resources, especially for essential medicines that cannot be substituted when stockouts occur.

Restricted facility spending authority further amplified the effects of limited and unpredictable revenue. All participating facilities required external authorisation to spend funds held in facility accounts, with reported approval timelines ranging from weeks to months. These delays were described as particularly consequential when central supplies were delayed or incomplete and facilities needed to respond quickly to commodity gaps. This finding is consistent with the governance tension within Kenya’s devolved system, where devolution sought to bring decision making closer to communities [17], yet the Public Finance Management Act of 2012 strengthened county level control over public funds and, in practice, often limited facility level decision space for routine operational decisions [22]. Our findings support the interpretation that the challenge is not only the adequacy of financing, but also the institutional rules that govern whether funds can be used when needed.

This represents a structural misalignment between decentralisation rhetoric and practical fiscal decision space at facility level. This contrasts with successful decentralized financing models, such as Thailand’s Universal Coverage Scheme, where district-level contracting units retain significant purchasing authority, enabling them to respond rapidly to local needs despite central budget caps [44, 45]. Evidence from other decentralised systems, including Colombia and Chile, similarly shows that the distribution of decision space across levels significantly shapes equity in resource allocation [34]. In the Kenyan context, it is critical to distinguish the loci of reform: while the National government determines the overarching legal framework (PFM Act) and reimbursement rates [16], County governments possess the administrative discretion to streamline authorization workflows and enact facility-level retention laws (Facility Improvement Fund Acts) that can bypass current bottlenecks [46, 47].

This study also adds to Kenyan literature on health financing under devolution, including work by Barasa, Tsofa and colleagues, and Kairu and colleagues, by focusing on the facility as the unit of analysis for chronic care delivery and integrating routine facility financial records with detailed accounts from facility in charges [15, 42]. While prior work has documented weaknesses in planning, budgeting, and accountability arrangements at county and facility levels, our contribution is to clarify how three elements combine at facility level to shape chronic care continuity. First, narrow and unstable revenue sources limit liquidity. Second, the absence of dedicated budgeting reduces visibility and prioritisation of hypertension and diabetes commodities. Third, centralised approval rules delay spending, which constrains the capacity to respond to supply gaps. We conceptualise this interaction as a facility level triple constraint that links financing architecture and governance rules to measurable interruptions in service readiness and medicine availability.

A critical insight from this study is that chronic care continuity depends as much on facility decision space as on funding volume. Facilities with trained staff, established clinical protocols, and patient demand nonetheless failed to maintain consistent hypertension and diabetes services because governance structures prevented timely resource use. Even when funds existed in facility accounts, whether from insurance reimbursements, user fees, or county allocations, centralized approval requirements created delays of weeks to months that translated directly into medicine stockouts and service interruptions. These findings challenge financing reform approaches that focus exclusively on increasing resource allocation without addressing the institutional rules that determine whether facilities can deploy resources effectively when needed. For chronic conditions requiring continuous medicine availability, procurement delays measured in weeks or months can precipitate treatment interruptions, patient loss to follow-up, and preventable complications, regardless of total funding levels. The governance implication is that reforms must address both resource adequacy and decision space constraints simultaneously to achieve sustained chronic care delivery.

The implications for ongoing reforms are timely. As Kenya transitions from NHIF purchasing arrangements toward the Social Health Authority framework within the universal health coverage agenda, the findings highlight priorities for implementation and evaluation [11, 48]. Purchasing reforms that improve payment timeliness and align reimbursement with the costs of delivering hypertension and diabetes care may improve facility liquidity, but continuity will remain vulnerable if approval rules continue to delay routine and emergency procurement. Similarly, as external partner support evolves, counties may need to strengthen predictable domestic allocations and establish practical mechanisms that allow accountable local procurement when central supplies are delayed. These findings therefore provide a baseline for future evaluation of whether Social Health Authority implementation improves reimbursement timelines, facility liquidity, approval turnaround time, and continuity of essential medicine availability for chronic care [9, 26].

### Study limitations

This study included seven facilities in one rural sub county, which limits statistical generalizability to other contexts in Kenya. However, the facilities covered the major public primary health care levels providing hypertension and diabetes services within the sub county, and the mixed methods approach strengthens the credibility of the identified mechanisms. The cross-sectional design captures a single time period and cannot establish causal relationships or trends over time. In addition, data collection occurred during the transition period when NHIF was still operational and before full implementation of the Social Health Authority, so financing arrangements may evolve. Future research should assess whether ongoing reforms improve facility autonomy, predictability of funding, and commodity availability for chronic care.

## Conclusion

Rural primary health care facilities in Kisumu County operated within financing arrangements that constrained effective hypertension and diabetes service delivery. Limited and fragmented revenue sources, absence of dedicated budgeting for chronic care, and centralised expenditure approval processes were associated with delays in procurement and frequent interruptions in essential medicine availability. Strengthening chronic care under universal health coverage will require reforms that expand accountable facility decision space, improve predictability of county disbursements, and ensure timely purchasing and reimbursement. As implementation of the Social Health Authority progresses and external partner support evolves, monitoring changes in payment timeliness, facility liquidity, and commodity availability will be essential to assess whether reforms improve continuity of chronic care. Ultimately, the sustainability of effective hypertension and diabetes management depends on expanding facility decision space, not only on increasing funding volume.

### Recommendations

Reforms requiring county-level action:

- Establish ring-fenced NCD budget lines in county and facility budgets (counties control budget formulation).
- Grant facilities financial autonomy through “spend-at-source” provisions or revolving funds (counties control facility expenditure authorization).
- Streamline facility expenditure approval processes through digital systems (counties control approval workflows).
- Increase direct county allocations to facilities from county equitable share revenues (counties control resource allocation to facilities).

Reforms requiring national-level action:

- Provide policy guidance and standardized frameworks for NCD budgeting across counties (national Ministry of Health coordination role).
- Ensure SHA provider payment mechanisms are timely and adequate (SHA is a national institution).
- Develop model county by-laws enabling conditional facility autonomy within PFM Act requirements (national Ministry of Health technical support).
- Establish national standards for facility financial autonomy with accountability safeguards (requires national policy framework).

Reforms requiring both county and national coordination:

- Monitoring and evaluation of SHA implementation effects on facility financing and service continuity (national SHA policy, county implementation).
- Capacity building for county health management teams on evidence-based NCD budgeting (national technical assistance, county implementation).
- Systematic costing studies to determine adequate NCD service financing levels (national research coordination, county-specific application).

Health Facility Managers:

- Evidence-Based Advocacy: Utilize facility-level data on NCD burden and stockouts to justify budget requests during the “bottom-up” planning cycle.
- Inventory Management: Strengthen local tracking of NCD commodity consumption to provide early warnings for potential stockouts before they reach a crisis point.

Researchers and implementation partners:

- Post-SHA Impact Studies: Conduct longitudinal research to evaluate how the transition from NHIF to SHA affects facility-level revenue and service readiness.
- Cost-Effectiveness Analysis: Perform a detailed costing study of NCD care at Level 2 and 3 facilities to provide data-driven benchmarks for future county budget allocations.

## Supplementary Information

Supplementary Material 1.

## Data Availability

The datasets generated and analyzed during this study are not publicly available due to confidentiality agreements with participating health facilities and ethical restrictions related to potentially identifiable facility-level financial data. However, anonymized datasets are available from the corresponding author on reasonable request and with appropriate ethical approval.The data collection instruments (structured questionnaire and semi-structured interview guide) used in this study are provided as Additional File 1.

## Abbreviations

BMC: BioMed Central
BP: Blood Pressure
CHMTs: County Health Management Teams
CHUs: Community Health Units
DM: Diabetes Mellitus
FIF: Facility Improvement Fund
GDP: Gross Domestic Product
HSSF: Health Sector Services Fund
HTN: Hypertension
JOOUST: Jaramogi Oginga Odinga University of Science and Technology
KEPH: Kenya Essential Package for Health
KEMSA: Kenya Medical Supplies Authority
KES: Kenyan Shillings
LMICs: Low- and Middle-Income Countries
MCP: Model Care Provider (or remove if not used)
NACOSTI: National Commission for Science, Technology and Innovation
NCDs: Noncommunicable Diseases
NGO: Non-Governmental Organization
NHIF: National Hospital Insurance Fund
PCNs: Primary Care Networks
PEN: Package of Essential Noncommunicable Disease Interventions
PFM: Public Finance Management
PHC: Primary Health Care
SHA: Social Health Authority
SHIF: Social Health Insurance Fund
TB: Tuberculosis
UHC: Universal Health Coverage
USD: United States Dollar
VLIR-IUC: Vlaamse Interuniversitaire Raad- Institutional University Cooperation
WHO: World Health Organization
