# Supplementary material for: Financial determinants of effective hypertension and diabetes care in rural primary health facilities in Kisumu, Kenya: a mixed-methods study
Source: BMC Public Health. 2026 Mar 11;26:1275. doi: 10.1186/s12889-026-26963-8 (PMC13094046; doi:10.1186/s12889-026-26963-8)
Supplement: Supplementary file 1 — Supplementary Material 1. [file 12889_2026_26963_MOESM1_ESM.docx]

**FACILITY FINANCING INTERVIEW GUIDE**

**Financial Determinants of Effective Hypertension and Diabetes Care in Rural Primary Health Facilities**

**Target Respondent:** Facility In-Charge
**Interview Duration:** 45-60 minutes
**Language:** English
**Recording:** Audio recording with consent

**INTRODUCTION SCRIPT**

"Thank you for agreeing to participate in this study. This interview will explore how financial resources flow to your facility and how these financing arrangements affect your ability to provide care for patients with hypertension and diabetes. There are no right or wrong answers. I am interested in understanding your experiences and perspectives. The interview will take approximately 45-60 minutes. Do I have your permission to audio-record our conversation?"

**SECTION A: SOURCES OF FUNDING FOR NCD SERVICES**

**Opening Question:**
A1. Could you describe the different sources of financial resources that support health services at this facility?

*Probes:*

- Which sources contribute most substantially to your facility's operations?
- Do any of these sources provide funds specifically designated for managing chronic diseases like hypertension or diabetes?

**Follow-up Questions:**

A2. In your experience over the past year, which funding sources have been most reliable in terms of timing and consistency of disbursement?

*Probes:*

- How predictable are the amounts you receive from each source?
- Have there been significant changes in funding levels from any source in recent years?

A3. Regarding NHIF reimbursements: How would you describe your facility's experience with insurance reimbursements for hypertension and diabetes care?

*Probes:*

- Approximately how long does it typically take from claim submission to payment receipt?
- Do delays in reimbursement affect your facility's ability to maintain NCD services? If yes, how?

A4. Does your facility generate any internal revenue through user fees or other mechanisms? If yes, what proportion of this revenue can be used for NCD-related expenses?

**SECTION B: PLANNING AND BUDGETING FOR NCD SERVICES**

**Opening Question:**
B1. Can you walk me through how your facility develops its annual work plan and budget?

*Probes:*

- Who participates in this planning process?
- What timeframe does your planning cover—annual, quarterly?
- Is there a standard template or format you must follow?

**Follow-up Questions:**

B2. When preparing your facility's budget, are non-communicable diseases like hypertension and diabetes explicitly considered as budget line items?

*Probes:*

- If yes, how do you determine what allocation is appropriate for NCD services?
- If no, how are NCD-related expenses handled within your overall budget?

B3. After you submit your facility's budget to the county health department, what typically happens to it?

*Probes:*

- Does the county inform you about the final allocation your facility will receive?
- How closely does the final allocation match what your facility requested?
- Can you provide an example from a recent budget cycle?

B4. In your assessment, does the current planning and budgeting process enable your facility to adequately address the needs of patients with hypertension and diabetes? Why or why not?

*Probes:*

- What would make the budgeting process more effective for supporting NCD care?
- Are there specific constraints in the budgeting system that affect NCD services?

**SECTION C: FINANCIAL AUTONOMY AND FUND MANAGEMENT**

**Opening Question:**
C1. Does your facility maintain its own bank account? If yes, can you describe who controls decisions about spending funds from this account?

*Probes:*

- Who are the authorized signatories?
- Can you approve expenditures directly, or must they be authorized by county officials?

**Follow-up Questions:**

C2. When your facility identifies a need to purchase supplies or equipment—for example, to address a stockout of diabetes medications—what process must you follow?

*Probes:*

- Do you require approval from the county before making such purchases?
- If approval is needed, approximately how long does authorization typically take?
- Can you describe a recent experience where you needed urgent approval?

C3. For funds that your facility receives from different sources, do they all flow through the same process, or are there different procedures depending on the funding source?

*Probes:*

- For example, are NHIF funds handled differently than county allocations?
- Do any funds bypass the county treasury and come directly to your facility?

C4. How would you characterize your facility's level of financial autonomy—that is, your authority to make spending decisions locally?

*Probes:*

- Has this level of autonomy changed over time? If yes, how?
- How does the current level of financial autonomy affect your facility's operations?

**SECTION D: RESOURCE ALLOCATION AND UTILIZATION FOR NCD CARE**

**Opening Question:**
D1. When funds become available to your facility, how do you prioritize different competing needs?

*Probes:*

- How do chronic disease services like hypertension and diabetes management fit within your prioritization?
- What factors influence these prioritization decisions?

**Follow-up Questions:**

D2. Can you describe the main categories of expenditure that your NCD budget must cover?

*Probes:*

- Medications and supplies?
- Diagnostic equipment and maintenance?
- Staff time and training?
- Patient education materials?

D3. How frequently does your facility experience stockouts of essential medicines for hypertension and diabetes?

*Probes:*

- When stockouts occur, what are usually the underlying causes?
- When you experience a stockout, can your facility use available funds to purchase medications locally to bridge the gap?
- If not, what prevents you from doing so?

D4. Does your facility have adequate diagnostic equipment for hypertension and diabetes care—blood pressure machines, glucometers, test strips?

*Probes:*

- When equipment breaks down or supplies run out, how easily can your facility replace or repair them?
- What financial barriers exist to maintaining functional diagnostic capacity?

**SECTION E: IMPACT OF FINANCING MECHANISMS ON NCD SERVICE DELIVERY**

**Opening Question:**
E1. In your view, how do the financial arrangements we've discussed affect the quality and continuity of care your facility can provide to patients with hypertension and diabetes?

*Probes:*

- Can you provide specific examples of how financing issues have affected patient care?
- Are there patients you've had to refer to higher-level facilities primarily because of resource constraints?

**Follow-up Questions:**

E2. Do delays in receiving funds or restrictions on how funds can be spent affect your staff's morale or ability to deliver care?

*Probes:*

- Can you describe how your clinical team responds when resources are inadequate?

E3. From the patient perspective, how do you think financing constraints affect their experience and outcomes?

*Probes:*

- Do patients face additional costs when your facility cannot provide services?
- Does this affect whether patients continue treatment or are lost to follow-up?

E4. Have you observed any changes in NCD service capacity at your facility over the past 2-3 years? If yes, what do you attribute these changes to?

*Probes:*

- Have funding patterns changed?
- Have policy changes affected your facility's financial situation?

**SECTION F: RECOMMENDATIONS FOR IMPROVEMENT**

**Opening Question:**
F1. Based on your experience managing this facility, what changes in financing arrangements would most improve your ability to provide effective care for patients with hypertension and diabetes?

*Probes:*

- Changes at the facility level?
- Changes at the county level?
- Changes in national policy or funding mechanisms?

**Follow-up Questions:**

F2. If you had greater control over your facility's financial resources, what would you do differently to strengthen NCD services?

F3. Are there examples from other facilities or counties that you think represent better approaches to financing NCD care at the primary level?

**CLOSING**

"Thank you very much for sharing your experiences and insights. Is there anything else about health facility financing or NCD care that you think is important for me to understand that we haven't discussed?"

"Do you have any questions for me about this study or how the information will be used?"

**OBSERVATIONAL CHECKLIST (Complete during or immediately after interview)**

**Document availability of the following (observe and note condition):**

☐ Blood pressure apparatus (manual and/or digital) - Functional? Adequate for patient load?

☐ Glucometers - Functional? Adequate for patient load?

☐ Test strips (glucose, urine) - Available? Adequate stock?

☐ Weighing scale - Functional?

☐ Height measuring tape - Available?

☐ Essential hypertension medications visible in pharmacy

- List observed: _______________

☐ Essential diabetes medications visible in pharmacy

- List observed: _______________

☐ National NCD guidelines (physical copy) - Visible in consultation area?

☐ Patient education materials (posters, pamphlets) - Visible?

☐ NCD patient register - Available for review?

**Facility infrastructure observations:**

- Consultation room privacy: _______________
- Waiting area adequacy: _______________
- General facility condition: _______________

**INTERVIEWER NOTES**

**Date of interview:** _______________
**Facility code:** _______________
**Interview start time:** _______________
**Interview end time:** _______________
**Interviewer name:** _______________

**Post-interview reflections:**

- Key themes that emerged:
- Notable quotes or observations:
- Areas requiring follow-up:
- Quality of rapport with respondent:

**Source and Adaptation Note:**
This interview guide was developed for this study, informed by the WHO health financing framework and adapted from approaches used in health facility financing assessments in devolved contexts (References: WHO Health Financing Framework; Kairu et al., 2021; Tsofa et al., 2017; Ouma et al., 2025). Questions were tailored to explore the specific intersection of financing mechanisms and NCD service delivery in rural primary health care settings under Kenya's devolved governance structure.

# Facility Financing Assessment Tool v2

**Instrument – Health Facility Abstraction Tool**

**SECTION 1: HEALTH FACILITY BACKGROUND**

| **(date)** Date of data collection: | __/__/____ [dd/mm/yyyy] |
| --- | --- |
| **(collector)** Data collector’s name: |  |
| **(facility)** Facility name: |  |

**PHC facility Location Information**

| **Q #** | **Variable name** | **Variable label** | **Information/ Code** |
| --- | --- | --- | --- |
|  | **project_id** | PHC facility ID (assigned by Study Team prior to data collection): |  |
|  | **county** | Name the County of the Study | [*Kisumu*] |
|  | **sub_county** | Name the Sub County of the Study | [*Seme*] |
|  | **type_location** | Facility location | Urban – 1, Semi-urban –2, Rural – 3 |

1. **Sources of Funds and Revenue Generation**

| **Q #** | **Variable name** | **Variable label** | **Code** |
| --- | --- | --- | --- |
| *B1* | **funding_sources** | What are the different sources of funding for this health facility in the FY 2023/24? |  |
|  | **funding_sources_amt** | From each of these sources, how much did you receive for the year? |  |
|  | **ncd_sources** | Does your facility receive any direct financial support from any source specifically for hypertension and diabetes care? |  |
|  | **ncd_sources_fr** | If yes, how frequently is this funding received? | Monthly  Quarterly  Annually  Irregularly(on request basis) |
| B2 | **revenue_ncds** | Does the facility generate revenue through user fees for services related to hypertension and diabetes? | Yes – 1, No – 0 |
| B2a | **revenue_ncds_how** | What percentage of the total funding for your facility is allocated specifically to the management of non-communicable diseases like hypertension and diabetes? | Less than 10%  11-25%  26-50%  More than 50%  Not specified |
| B3 | **funding_changes** | Has your facility experienced any changes in funding for hypertension and diabetes care over the last 2-3 years? | Yes, increased  Yes, decreased  No change  Don’t know |
|  | **funding_changes_why** | If yes, **what were the reasons for the changes?** (Select all that apply) | New funding from county government  Reduced donor funding  Increased out-of-pocket payments  Expanded NHIF coverage  Other (Please specify): __________ |
|  | **funding_challenges** | What challenges does your facility face in securing adequate funding for managing hypertension and diabetes? (Select all that apply) | Inconsistent government funding  Limited donor funding  Lack of financial autonomy at the facility level  High out-of-pocket costs for patients  Insufficient NHIF reimbursement  Other (Please specify): __________ |

1. **Planning and budgeting**

| **Q #** | **Variable name** | **Variable label** | **Code** |
| --- | --- | --- | --- |
| *A1* | **work_plan** | Does this facility prepare annual work plans? | Yes – 1, No – 0 |
| A1b | **planning_processs** | Could you describe the facility planning process? What is the purpose of the planning? What do you plan for?  Is there a prescribed template for planning? Who is involved? Is this done annually, quarterly, or at other intervals? | text |
| A2 | **bedgeting** | Does your facility prepare budgets for its services, including hypertension and diabetes care? | Yes – 1, No – 0, Unable to determine – 9 |
| A2b | **budgeting_process** | Can you describe the budgeting process? What does the budget cover (e.g., staff, medications, equipment)?  Who is involved in preparing the budget? Is there a standard format you follow? | text |
| A3 | **ncds_considered** | Are non-communicable diseases like hypertension and diabetes considered in the budget? | Yes, No |
| A3b | **ncds_service_cd** | What specific areas of hypertension and diabetes care are budgeted for (e.g., medications, diagnostic tools, staffing)? |  |
| A4 | **ensure_coverage** | How does your facility ensure that the budget adequately covers the needs of hypertension and diabetes care?  *Probe: Are there any specific strategies to make sure NCD care is prioritized?* |  |
| A5 | **recuring_issues_dsb** | Are there recurring issues with the availability of funds for hypertension and diabetes care? Can you describe these issues? How do they impact service delivery? | Yes – 1, No – 0, Unable to determine – 9 |
| A5b | **opinion_plan** | In your opinion, is the current planning and budgeting process effective in helping the facility provide adequate care for hypertension and diabetes? | **text** |

1. **Flow of funds**

| **Q #** | **Variable name** | **Variable label** | **Information/ Code** |
| --- | --- | --- | --- |
| C1 | **bank_account** | Does the facility operate a bank account? | Yes – 1, No – 0 |
| C2 | **dep_facility_accnt** | For each of the sources of funds that you have listed above, are the funds deposited to the facility bank account directly? | Yes – 1, No – 0, Unable to determine – 9 |
| C2a | **dep_other_accnt** | If they are not deposited directly, where are the funds sent to? | If c2 is No |
| C2b | **access_other_accnt** | How do you access funds that are not sent directly to your facility bank account? | If c2 is No |
| C3 | **autonomy** | For the funds that are in your account, are you allowed to spend directly from the account? | If c1 ==yes  Yes – 1, No – 0, Unable to determine – 9 |
| C4 | **approval** | Do you need any permission/approvals to spend these funds? | Yes – 1, No – 0, Unable to determine – 9 |
| C4a | **approval_who** | If yes, **who grants the approval?** | If c8 = yes  text |
| C4b | **approval_duration** | On average, how long does it take for you to get the authorization to spend funds? | 1-2 weeks?  3-4 Weeks  2-3 months  More than 3 months  Other |

1. **Financial Utilization for NCD Care**

| **Q #** | **Variable name** | **Variable label** | **Information/ Code** |
| --- | --- | --- | --- |
|  | **staff_train** | **Does your facility have staff trained in NCD care?** | **Yes, No** |
| D3 | **equip_available** | Does the facility have diagnostic equipment (BP machines, glucose monitors) | Yes, no |
| D3a | **equip_adequate** | Is the available diagnostic equipment (BP machines, glucose monitors) adequate for the current patient load? | Yes, no |
| D4 | **stockouts** | How frequently does your facility experience stockouts of hypertension and diabetes medications? |  |
| D4a | **stockouts_why** | What are the main causes of stockouts? |  |
| D4 | **perception_effective** | How effective are the following in supporting hypertension and diabetes care?  NHIF reimbursements  County government allocations  External donor funding  Facility revenue from user fees | Very effective  Effective  Somewhat ineffective  Ineffective |
| D4a | **effective_nhif** | NHIF/SHIF reimbursements |  |
| D4b | **effective_county** | County government allocations |  |
| D4c | **effective_donor** | External donor funding |  |
| D4d | **effective_userfee** | Facility revenue from user fees |  |
|  |  | Is there delay |  |
| D4 | **delay_impact** | How does the delay or timing of funds affect service delivery for hypertension and diabetes (e.g., delayed purchasing of medications, delayed staff payments)? |  |
| D5 | **specific_chall** | Are there specific financial challenges related to hypertension or diabetes (e.g., higher costs for insulin, need for diagnostic machines)? |  |
| D5a | **specific_chall_ad** | If so, how does the facility handle these? |  |
| D6 | **fin_chall_qual** | How do financial challenges affect the quality of care for NCD patients (e.g., medication stockouts, understaffing)? |  |
| D7 | **barriers** | What are the biggest barriers to securing adequate funding for hypertension and diabetes care? |  |
| D8 | **recommendation** | Please provide recommendations for improving the financial management of hypertension and diabetes care at both the facility and county levels. |  |
|  |  | In your experience, how have the different sources of funding (e.g., NHIF, County Government, user fees) influenced the quality and continuity of care for hypertension and diabetes patients?   - Probe: Can you share specific examples where funding helped or hindered service delivery? |  |

**END OF DATA COLLECTION INSTRUMENTS**
